# Detection of a MicroRNA Signal in an *In Vivo* Expression Set of mRNAs

**DOI:** 10.1371/journal.pone.0000804

**Published:** 2007-08-29

**Authors:** Tsunglin Liu, Thales Papagiannakopoulos, Kathy Puskar, Shuping Qi, Fernando Santiago, William Clay, Kaiqin Lao, Yohan Lee, Stanley F. Nelson, Harley I. Kornblum, Frank Doyle, Linda Petzold, Boris Shraiman, Kenneth S. Kosik

**Affiliations:** 1 Neuroscience Research Institute, University of California at Santa Barbara, Santa Barbara, California, United States of America; 2 Department of Molecular, Cellular and Developmental Biology, University of California at Santa Barbara, Santa Barbara, California, United States of America; 3 Kavli Institute of Theoretical Physics, University of California at Santa Barbara, Santa Barbara, California, United States of America; 4 Department of Computer Science, University of California at Santa Barbara, Santa Barbara, California, United States of America; 5 Department of Chemical Engineering, University of California at Santa Barbara, Santa Barbara, California, United States of America; 6 Department of Human Genetics, David Geffen School of Medicine, University of California at Los Angeles, Los Angeles, California, United States of America; 7 Departments of Psychiatry, Molecular and Medical Pharmacology, the Semel Institute and the Jonsson Comprehensive Cancer Center, David Geffen School of Medicine, University of California at Los Angeles, Los Angeles, California, United States of America; 8 Department of Mathematics, Jackson State University, Jackson, Mississippi, United States of America; 9 Applied Biosystems, Foster City, California, United States of America; Institute of Genetics and Molecular and Cellular Biology, France

## Abstract

**Background:**

microRNAs (miRNAs) are approximately 21 nucleotide non-coding transcripts capable of regulating gene expression. The most widely studied mechanism of regulation involves binding of a miRNA to the target mRNA. As a result, translation of the target mRNA is inhibited and the mRNA may be destabilized. The inhibitory effects of miRNAs have been linked to diverse cellular processes including malignant proliferation, apoptosis, development, differentiation, and metabolic processes. We asked whether endogenous fluctuations in a set of mRNA and miRNA profiles contain correlated changes that are statistically distinguishable from the many other fluctuations in the data set.

**Methodology/Principal Findings:**

RNA was extracted from 12 human primary brain tumor biopsies. These samples were used to determine genome-wide mRNA expression levels by microarray analysis and a miRNA profile by real-time reverse transcription PCR. Correlation coefficients were determined for all possible mRNA-miRNA pairs and the distribution of these correlations compared to the random distribution. An excess of high positive and negative correlation pairs were observed at the tails of these distributions. Most of these highest correlation pairs do not contain sufficiently complementary sequences to predict a target relationship; nor do they lie in physical proximity to each other. However, by examining pairs in which the significance of the correlation coefficients is modestly relaxed, negative correlations do tend to predict targets and positive correlations tend to predict physically proximate pairs. A subset of high correlation pairs were experimentally validated by over-expressing or suppressing a miRNA and measuring the correlated mRNAs.

**Conclusions/Significance:**

Sufficient information exists within a set of tumor samples to detect endogenous correlations between miRNA and mRNA levels. Based on the validations the causal arrow for these correlations is likely to be directed from the miRNAs to the mRNAs. From these data sets, we inferred and validated a tumor suppression pathway linked to *miR-181c*.

## Introduction

miRNAs regulate both the stability and translatability of their mRNA targets [1]. This regulation occurs by binding of the ∼21 nucleotide mature miRNA to an imperfectly matched sequence within the target mRNA in animals. Most descriptions of miRNA function focus either on their role as post-transcriptional regulators of target mRNAs or at a much higher level on their cell biological processes and organismal roles. Between these two roles lies a poorly understood landscape through which the miRNA signal must travel to implement its cell biological function at the phenotypic level. Following reductions in the level of a protein encoded by a target mRNA, subsequent effects may alter the levels of other mRNAs and thus changes in miRNA expression will ramify through the transcriptional profile. As the networked link to miRNA fluctuation becomes more distant and diluted, correlative changes in mRNAs may no longer be discernable among the many other factors that control change in mRNA levels across a set of transcriptional profiles. However, changes in mRNAs that may not be direct targets, but lie closer to the consequences of miRNA inhibitory effects may be discernable in the transcriptional profile.

Often the effects of miRNAs on mRNAs are studied by expressing or suppressing the miRNA in cells. Because it is very likely that non-physiological levels of miRNAs will alter the target field, i.e. the specific set of mRNAs targeted, a more accurate measure of miRNA effects requires endogenous determinations. To discover cases in which miRNAs exert detectably strong relationship to the level of an mRNA within a set of transcriptional profiles we collected a set of human glioma samples and performed broad-scale analyses of miRNA levels and mRNA levels. Broad based gene expression in cancer data sets, which are known to vary in a manner that is predictive of tumor grade and patient prognosis [2]–[4], were used to find significant correlations between endogenous fluctuations in the miRNA and mRNA expression.

mRNAs were measured with oligonucleotide arrays (Affymetrix). The miRNA profile was measured by multiplex RT-PCR (Applied Biosystems) which can discern the specificity of miRNAs even for very similar sequences from the same family [5]. These methods are more reliable when comparing the expression of the same miRNA, which was done in this study. The statistical analysis applied to the data set revealed an excess of high correlation miRNA-mRNA pairs beyond the number expected by chance. A subset of these high correlation pairs was experimentally validated and revealed a network of linked genes related to carcinogenesis.

## Results

### Detection of highly correlated of miRNA-mRNA pairs

We measured the expression levels of 330 miRNAs and about 14,500 genes in 12 brain tumor biopsies from humans using multiplex RT-PCR and Affymetrix oligonucleotide microarrays, respectively. Before correlation analysis, we filtered data with substantial noise due to low abundance in order to gain more confidence in the resulting correlations (see Methods). We also filtered those data with little variation across the 12 samples because the absence of high variation will result in correlations mainly due to noise. These criteria left 93 miRNAs and 7,089 probe sets for the analysis. This approach was chosen because we intended to ask whether there existed significant correlation within a highly accurate data set, instead of extracting lower reliability data from the entire data set. Both the mRNA and the miRNA data sets were normalized by quantile normalization (see Methods). After normalization, we calculated the Pearson's correlation between the expression profiles of the miRNAs and the mRNAs, and compared these correlations with the random case. The random case is referred to as any result obtained from randomly generated or permuted expression profiles. Here, we asked whether the distribution of 93*7,089 = 659,277 correlation coefficients, r's, from the data set was significantly different from the random case.

**Figure 1:**
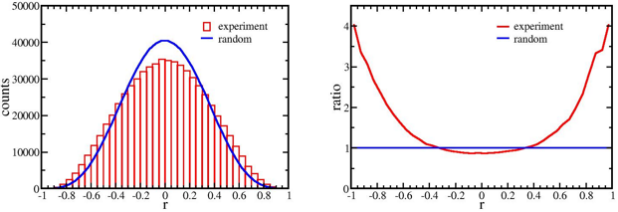
Distribution of correlation coefficients. (A) The histogram consists of 40 bins ranging from −1 to 1. (B) The ratio of number of correlation coefficients in the experimental data to the random case for bin in Figureô 1A. At high correlations, the number of microRNA-mRNA pairs is larger than expected in the random case. This result suggests information exists in the correlation coefficients between miRNAs and mRNAs.

The distribution of r's between our data set and the random case (see Methods for a derived closed form solution of the random case) clearly differed (Figure 1A). To ensure a truly random control, we randomly permuted the order of samples in miRNA (or mRNA) profiles and repeated the analysis 100 times. Such random permutations preserved the autocorrelations among miRNAs and mRNAs, and generated results close to the random case. Quantitatively, our data set output 74 and 1631 correlations below −0.9 and −0.8 respectively, while the output from the random cases was 23.65 and 615.12 on average. The binomial probability to obtain at least 74 and 1631 correlations from 659277 random trials with a probability of success (23.65/659277) and (615.12/659277) was 1.4*10^−11^ and 1.6*10^−11^ respectively. Similarly, the binomial probability to obtain at least 75 and 1735 correlations above 0.9 and 0.8 from 659277 random trials with a probability of success (23.73/659277) and (628.19/659277) was 3.6*10^−11^ and 1.1*10^−11^ respectively. These small p-values indicated the significant difference between our data set and the random cases. Thus, the correlations between miRNAs and mRNAs contained information regarding interactions, but did not suggest a causal direction of the interaction. The difference between the distribution of correlations in the data set and that of the random distributions, was re-plotted with the ratio between the number of r's in each range of experimentally derived coefficients to the random case (Figure1B). At both positive and negative correlation tails, the miRNA-mRNA correlations were stronger and more numerous than expected in the random case.

As seen in Figure1B the high positive and negative correlations at the tails indicated a set of miRNA-mRNA correlations that were stronger and more numerous than expected in the random case. To define this set we defined a cutoff r_c_ according to the equality
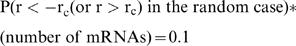
This says that for a given miRNA, the expected number of mRNAs with correlation greater than r_c_ (∼0.916 with 7089 mRNAs) is only 0.1 in the random case. A correlation coefficient was considered high when the absolute value was above the 0.916 cutoff. With a total of 93 miRNAs, we would expect 9.3 pairs with a high negative correlation by chance; however we obtained 35 such pairs from our experimental data (Table1), of which 27% were expected to occur by chance. Similarly, for the positive correlations, we obtained 37 such pairs, of which 25% were expected to occur by chance. In other words, approximately one quarter of the total high correlation pairs—72 positive and negative correlations (Table1)—could be false positives. Nevertheless, the binomial probability in obtaining at least 72 coefficients above the cutoff with the probability of success 0.1 is very low (P∼5*10^−21^).

**Table 1:** List of miRNA-mRNA high correlation pairs and number of predicted target sites.

miRNA(hsa-)	mRNA	r	site	miRNA(hsa-)	mRNA	r	site	miRNA(hsa-)	mRNA	r	site
*let-7b*	*ULK2*	0.93	1	*miR-195*	*SF3B2*	0.92	0	*miR-30d*	*ASTN*	0.94	0
*let-7i*	*CBLB*	−0.92	0	*miR-195*	*AARS*	0.93	0	*miR-30d*	*TAOK3*	0.94	0
*miR-106b*	*ADSS*	−0.92	0	*miR-195*	*SVIL*	−0.94	1	*miR-340*	*LAMC1*	−0.96	0
*miR-106b*	*TCF4*	0.96	0	*miR-195*	*LNK,SH2B3*	−0.92	0	*miR-340*	*LPHN2*	−0.93	0
*miR-139*	*INPP5A*	0.96	0	*miR-195*	*MAPT*	0.92	0	*miR-340*	*DNM3*	0.94	0
*miR-142-5p*	*SNAP23*	0.92	1	*miR-195*	*FTO*	0.93	0	*miR-342*	*LPHN1*	−0.92	0
*miR-142-5p*	*EVI2B*	0.92	1	*miR-195*	*SCN3A*	0.92	1	*miR-342*	*APC2*	−0.92	0
*miR-181c*	*PCAF*	0.93	1	*miR-19a*	*ANXA4*	−0.93	1	*miR-365*	*APIP*	0.92	0
*miR-181c*	*ASCC1*	0.95	0	*miR-19a*	*GSTO1*	−0.92	0	*miR-92*	*ANXA7*	−0.93	0
*miR-193a*	*TM9SF1*	0.92	0	*miR-19a*	*IFITM1*	−0.94	1	*miR-182*	*FADS2*	0.92	0
*miR-193a*	*B3GALNT1*	0.94	0	*miR-19a*	*DDX11*	0.93	0	*miR-182*	*HPS1*	−0.93	0
*miR-193a*	*SOD2*	0.92	0	*miR-19a*	*TTC3*	0.93	0	*miR-204*	*HRASLS*	−0.92	0
*miR-193a*	*PBEF1*	0.93	0	*miR-19a*	*ENTPD1*	−0.93	0	*miR-9*	*ATP6V0E*	−0.93	0
*miR-194*	*AARS*	0.95	0	*miR-19a*	*NCF2*	−0.92	0	*miR-9*	*AMPD3*	−0.93	0
*miR-194*	*LNK,SH2B3*	−0.96	0	*miR-19a*	*PRR13*	−0.92	0	*miR-9*	*CCNG1*	−0.92	1
*miR-194*	*REEP1*	0.93	0	*miR-27a*	*PDZD8*	−0.92	0	*miR-99a*	*MAPT*	0.93	0
*miR-194*	*INA*	0.92	0	*miR-27b*	*FSCN1*	0.92	0	*miR-99a*	*MAPT*	0.92	0
*miR-194*	*LY75*	−0.95	0	*miR-29a*	*TRIM37*	−0.94	2	*miR-155*	*RGS17*	0.95	0
*miR-194*	*PBX1*	0.92	0	*miR-29b*	*DDX50*	0.94	0	*miR-155*	*SPRY4*	0.92	0
*miR-194*	*SCAMP5*	0.92	0	*miR-30b*	*RAP2C*	−0.93	2	*miR-376b*	*TULP3*	−0.92	0
*miR-194*	*RPIP8*	0.92	0	*miR-30c*	*TXNIP*	−0.93	0	miR-518a-2*	*COPS4*	−0.93	0
*miR-194*	*DLST,DLSTP*	−0.95	0	*miR-30c*	*ULK2*	−0.92	0	miR-518a-2*	*DCHS1*	−0.92	0
*miR-194*	*CTBS*	−0.94	0	*miR-30d*	*TSPAN7*	0.92	0	*miR-93*	*PVRL2*	−0.93	0
*miR-194*	*C1orf78*	−0.95	0	*miR-30d*	*PFKM*	0.95	0	*miR-95*	*HS2ST1*	−0.96	0

The clear correlation signal arising from just 12 samples was somewhat surprising given the very large number of correlations tested. However, the finding was validated by demonstrating a decline in excess strong correlations over the random case with fewer samples (Figure S2, where the ratio in number of coefficients, similar to Figure1B, was plotted). The signal declined with fewer samples because the correlation between a miRNA and an mRNA in the actual data did not increase as fast as the probability for high correlations in the random case. This difference from the random case increased our confidence that the signal was bona fide.

### Target Predictions among Correlated Pairs

Among the explanations for high negative correlation pairs is direct targeting of the mRNA by the paired miRNA. Therefore, we checked the percentage of miRNA-mRNA high correlation pairs that were predicted as target pairs by TargetScan [6]. Among the 35 high negative correlation pairs four were predicted to have one target site (Table1). This number of predicted targets may represent chance because among 37 high positive correlation pairs five were predicted to have one target site. No high positive correlation pair has two predicted sites, but among the high correlation negative correlation pairs two pairs (in addition to the four single target site pairs) had two predicted target sites (Table1). Among the 659277 miRNA-mRNA pairs, 84238 (∼12.8%) were predicted targets. Thus, with 35 (or 37) pairs, we would expect about 4∼5 predicted target pairs by chance, which was the case for the highly correlated miRNA-mRNA pairs. These findings demonstrate that the dominant feature of the miRNA-mRNA relationship in terms of the magnitude of change in the mRNAs is indirectly related to the binding of the miRNA to its mRNA targets. miRNA binding to a target usually has only a modest effect on the level of the target mRNA and a greater effect on the encoded protein. Therefore, direct binding effects are more difficult to detect in an endogenous data set of profiles. However, the ability to detect specific endogenous changes in an mRNA profile associated with miRNA fluctuations has not been previously reported.

**Figure 2:**
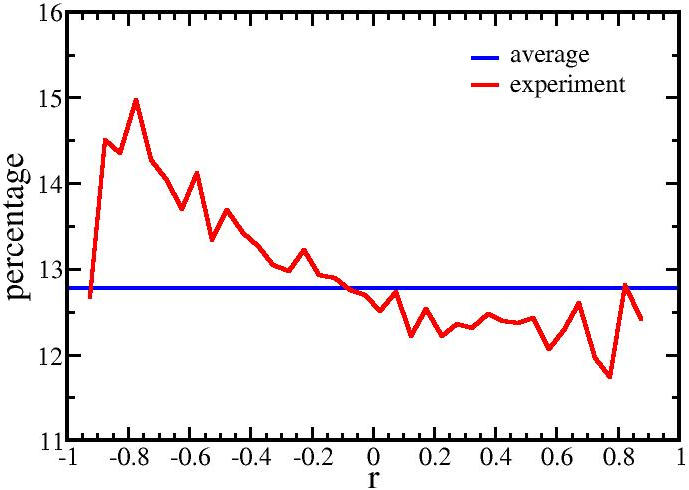
Percentage of predicted targets across a range of correlation coefficients. The percentage of targets over the range of correlation defined in Figureô 1 decreases with the increasing correlation between miRNAs and mRNAs. This implies that the correlation indeed captures information about target prediction, and the negative slope might be related to direct targeting effect between miRNAs and mRNAs.

Although most of the strongest miRNA-mRNA correlations are not likely to be due to direct targeting, it is possible to detect in the data set a weak effect that is consistent with miRNA targeting to specific mRNAs. The coefficients of all miRNA-mRNA pairs were split into 40 bins, ranging from −1 to 1 with a bin size 0.05 and the percentage of predicted target pairs for each bin calculated (Figure2). A clear trend was observed as the correlation went from negative to positive: the percentage of predicted target pairs decreased. As a control, we repeated the target analysis for the 100 random permutations mentioned above and compared their slopes (see Methods). None of the 100 random permutations gave a slope as negative as our observation, which indicated the significance of the trend. This finding is consistent with data that miRNAs can down regulate their targeted mRNAs [7], and therefore more predicted target pairs should lie in the region of negative correlation. This trend was apparent in the data even though about 87% of the miRNA-mRNA pairs were not predicted as target pairs by TargetScan.

**Figure 3:**
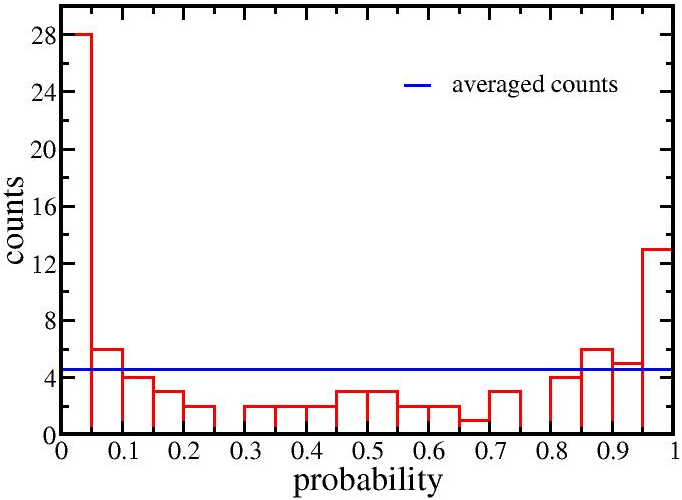
Histogram of P values from the WRS test for each of the miRNAs. The WRS test compared the correlations of the predicted targets with that of the non-predicted ones for each of the 91 miRNAs. As the correlation went from negative to positive the percentage of predicted target pairs decreased. Interestingly, some highly positively correlated miRNAs had high P values, which suggests an indirect relationship with the predicted target.

Having detected a relationship between negative correlation values and the percentage of predicted targets, we next asked whether for a given miRNA, do its predicted targets show more negative correlations. To this end, we split the 7,089 correlations of each miRNA into two groups, those with and without predicted targets. We then applied one sided Wilcoxon Rank Sum (WRS) test to check the null hypothesis, that the correlation of the first group is not lower than the second group. For each miRNA, the one sided WRS test gave a P-value, and a P-value smaller than a given cutoff indicated that the correlation with the predicted targets was more negative than that with non-predicted targets. Of 91 miRNAs (two miRNAs from the set of 93 were not in TargetScan.), 28 (∼30%) miRNAs fell below the significance level P = 0.05 (Figure3). Using the Bonferroni correction for multiple tests, 7 miRNAs were found to show significantly more negative correlations with the predicted targets, while only two were found to show more positive correlation (Figure3). No relationship was found between miRNA abundance and this difference in the rank sum. Thus, those pairs which are predicted targets, but showed a more positive correlation may be indirectly related.

### Positive Correlations between Proximate Pairs

Some miRNAs are intronic, and these miRNAs show correlated expression patterns with their host genes [8]. This observation was replicated in our data where almost all the miRNAs and their host genes showed positive correlations (Table2). Three miRNA-mRNA pairs, *miR-25/MCM7, miR-15b/SMC4L1*, and *miR-30c/NFYC* also appeared in the previous study. The reported correlation coefficients were 0.838, 0.509, and 0.406, respectively, values which are consistent with our data (Table2). We also observed the co-expression of neighboring miRNAs as reported in the same study (Figure S1).

**Table 2:** Correlations between intronic miRNAs and their host genes.

miRNA(hsa-)	Gene	r
*miR-106b*	*MCM7*	0.75
*miR-9*	*C1orf61*	0.73
*miR-140*	*WWP2*	0.61
*miR-25*	*MCM7*	0.6
*miR-15b*	*SMC4L1*	0.47
*miR-30e-3p*	*NFYC*	0.4
*miR-151*	*PTK2*	0.36
*miR-93*	*MCM7*	0.33
*let-7f*	*HUWE1*	0.33
*miR-30e-5p*	*NFYC*	0.31
*miR-30c*	*NFYC*	0.21
*miR-340*	*RNF130*	0.15
*miR-342*	*EVL*	0.06
*let-7g*	*TMEM113*	−0.06
*miR-214*	*DNM3*	−0.52

**Figure 4:**
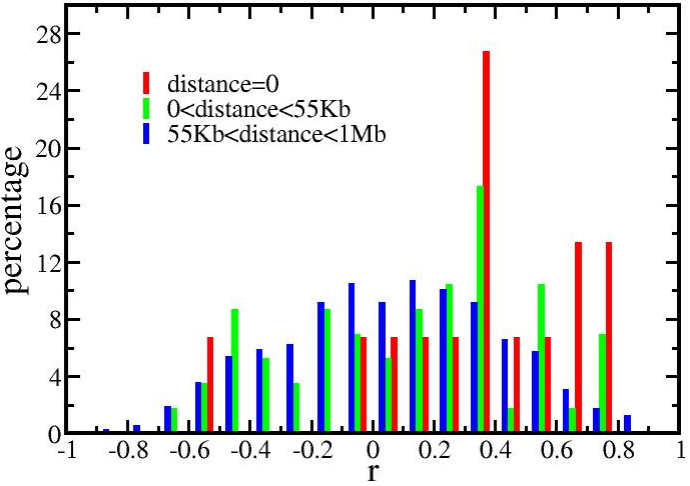
Distribution of correlations between miRNAs and proximate mRNAs. This figure showed the distributions for 3 groups of miRNA-mRNA pairs, classified by the distance between a miRNA and an mRNA on the same chromosome. The y-axis shows the percentage of miRNA-mRNA pairs in each range of correlation (−1 to 1 with binsize 0.1). An intronic miRNA had a distance zero to its host gene. The average length of a human gene, 55Kb (http://www.ncbi.nlm.nih.gov/Web/Newsltr/Spring03/human.html), was chosen as one distance boundary. As the distance between the correlated pair increased, the distribution increasingly shifted away from positive correlations to a normal distribution.

A positive correlation signal was also detected between mRNAs and those miRNAs that were close to, but not within the gene encoding the mRNA (Figure4). Although weak, the positive correlation signal persisted beyond the 1 Mb shown in the figure. Thus, co-expression comprises part of the positively correlated signal. However, the strongest co-expression signal from the intronic miRNAs and the host genes did not contain a correlation greater than 0.8 (Figure4). Therefore, proximity did not explain the very highly positive correlations.

### Validation of High Correlation Pairs

Three miRNAs (*miR-181c, miR-182, miR-19a*) were selected from among the miRNA-mRNA high correlation pairs to test whether the correlations predicted changes that could be reproduced experimentally. *miR-181c* and *miR-182* were individually transfected as pre-miRNAs into U251 glioblastoma cells to increase their levels 2–4 fold (data not shown). *miR-19a* was downregulated by addition of 2′*-O-*methyl (2′*-O-*Me)-*miR-19a*. The U251 cells were derived from a high-grade glioblastoma; therefore, they represent a reasonable cell culture system for experimental validations of data derived from glioblastoma biopsies. We determined whether the effects of each miRNA on the mRNAs with which it was highly correlated corresponded to the relationship predicted in the correlation data set. These experiments also served to suggest the direction of causality between the correlated pair.

**Figure 5:**
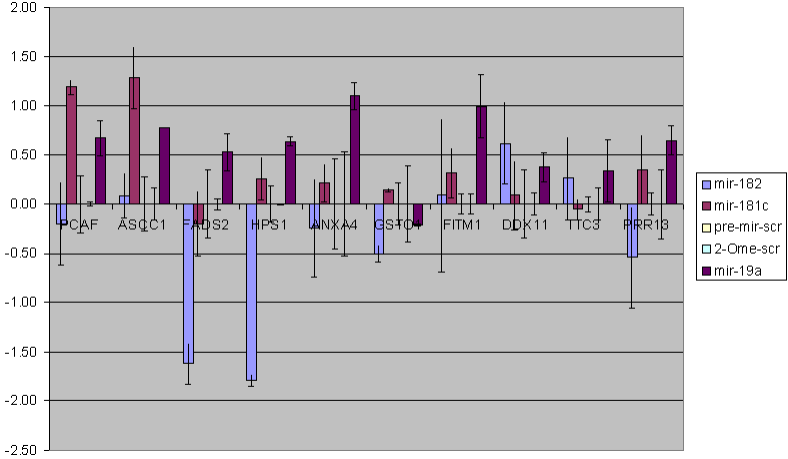
Experimental Validation of Highly correlated miRNA/transcript pairs. The above figure represents Ct_scrambled_-Ct_pre-mir/2′*-O-*Me_ from three individual transfection experiments of pre-*miR-181c*, pre-*miR-182*, 2′*-O-*Me-*miR-19a*, 2′*-O-*Me-scrambled and pre-mir-scrambled. *PCAF, ASCC1, FADS2, HPS1, ANXA4, GSTO1, IFITM1, DDX11, TTC3* and *PRR13* levels were determined after transfection.

Twenty-four hours after the transfections of pre-*miR-182*, pre-*miR-181c*, 2′*-O-*Me-*miR-19a*, as well as a pre-miR scramble or a 2′*-O-*Me scramble, we collected total RNA for real-time RT-PCR to determine the relative changes in the levels of the correlated mRNAs (Figure5). We computed a delta Ct by taking the difference in the mRNA values in cells exposed to the precursor miRNA or the 2′*-O-*Me-miRNA and cells exposed to the appropriate scrambled control. As negative controls we determined the levels of ten mRNAs that did not show any significant correlation with the miRNA being tested. For each miRNA, the expected outcomes are shown in the left box of Table3 as either positive (+) or negative (−) correlations based on the high correlation pairs in Table1, or no change (blank space) because the measured mRNA is not highly correlated with endogenous fluctuations in the test miRNA. The actual outcome of the experiment, i.e. change in mRNA level relative to the scrambled, is shown in the right box (Table3). Up or down changes of more than two-fold in the mRNA level are indicated by an up or down arrow; a difference less than two fold was denoted by a blank space. Most boxes represent control cases in which no significant correlation existed in the data set, nor did any change in the mRNA occur in the experiment (Table3). Thus, all of these cases bore out the predictions. Five cases with predicted correlative changes were borne out by experiment. In four cases, the data set predicted a correlation that did not occur experimentally and in one case the experiment and the prediction went in opposite directions (Table3). Given that we expect 26% to be false positives and the imprecision of U251 cells to replicate the in vivo fluctuations among the more diverse tumor samples this result indicates a very high degree of predictability.

**Table 3:** Validation of highly correlated pairs.

	miR-182 (⇑)		miR-181c (⇑)		miR-19a (⇓)	
PCAF			+⇑	⇑		
ASCC1			+⇑	⇑		
FADS2	+⇑	⇓				
HPS1	-⇓	⇓				
ANXA4					-⇑	⇑
GSTO1					-⇑	
IFITM1					-⇑	⇑
DDX11					+⇓	
TTC3					+⇓	
PRR13					-⇑	

The left box under each miRNA column indicates a high positive (+) or negative (−) correlation (**|r|>**0.916) of that miRNA with the mRNA in each row. The up or down arrow next to the plus or minus sign indicates the expected direction when the miRNA at the top of the column is experimentally changed. miR-182 and 181c were upregulated (⇑) and miR-19a was downregulated (⇓). The observed direction of change is indicated in the right box under each miRNA column. A difference of less than two fold is considered as no change and denoted by a blank.

Among the validated high correlation pairs was a positive correlation between *miR-181c* and *p300/CBP-associated factor* (*PCAF*). *PCAF* is a histone acetyltransferase that induces transcriptional activation by histone *H3* acetylation of target promoters [9]. Additionally, *PCAF* can acetylate lysines on non-histone proteins such as *tumor protein p53* and *retinoblastoma 1*, activating their tumor suppressor activity [10], [11]. Abrogating *PCAF* function by lowering its transcript levels is a mechanism by which the transforming growth factor-beta, retinoblastoma, and *p53* tumor suppressive pathways are negatively modulated in cancer. The validation experiment suggested that the direction of causality very likely went from *miR-181c* to *PCAF* (although if the pair is networked as a negative feedback loop in cis, causality *in vivo* could be bi-directional).

**Figure 6:**
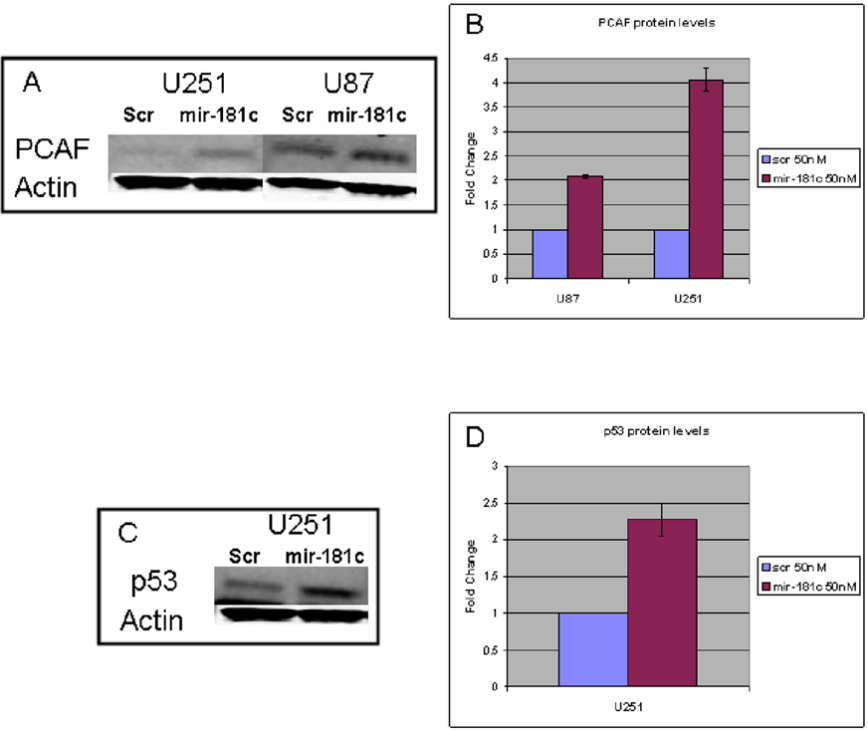
Immunoblot analysis of *miR-181c* treated cells. Immunoblots of U251 and U87 transfected cells transfected with either *miR-181c* pre-miRNA or a scrambled sequence. *PCAF* (A) and *p53* (C) were immunodetected and actin was used as a loading control. Quantification of the immunoblots showed a significant difference in B and C (p<0.01, t-test in all three comparisons).

We performed western blot analysis to verify the increase of *PCAF* protein levels in response to *miR-181c* upregulation in U251 cells and in an additional glioma line, U87. Twenty-four hours post-transfection we observed a significant increase in *PCAF* in the pre-*miR-181c* treated U87 and U251 cells when compared to the negative control scramble (Figure6A,B). This observation suggested that the increase of *PCAF* observed in the qPCR experiments was translated into an increase in the protein/enzyme levels in both glioma lines. The upregulation of *PCAF* in response to increased *miR-181c* could occur through a known transactivator of *PCAF*, *p53* [12], [13]. We checked the levels of *p53* in both U251 and U87 treated cells and observed a significant increase of *p53* protein levels in the *miR-181c* treated U251 cells (Figure6C,D); however we could not detect *p53* in the U87 cells.

**Figure 7:**
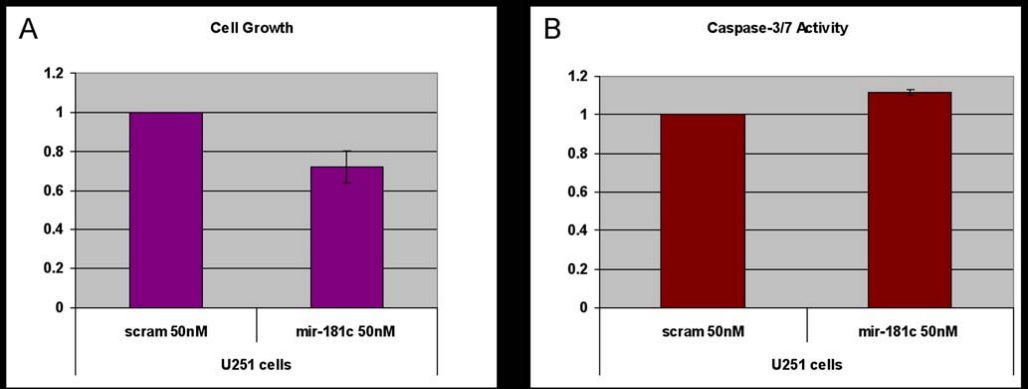
Cell Growth and Apoptosis Assays. U251 cells were treated with pre-*miR-181c* and negative control scramble. 24 hrs post-transfection, a) cell growth (p<0.01, t-test) and b) apoptosis were assayed (p<0.05, t-test).

**Figure 8:**
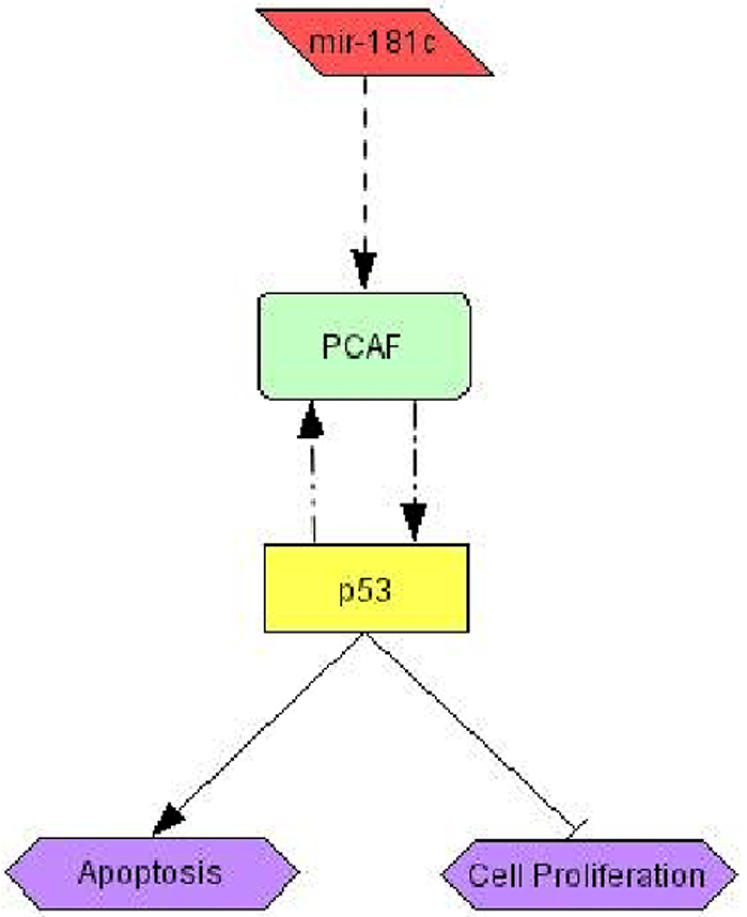
Tumor suppressive pathway involving *miR-181c.*

We then tested how *miR-181c* upregulation affected cell proliferation and apoptosis, because the upregulation of *PCAF* and *p53* protein levels in U251 cells could lead to activation of a plethora of other downstream tumor suppressive genes that can suppress the oncogenic state of these glioma cells. We used the Cell-Titer Glo assay to determine the number of metabolically viable cells upon addition of *miR-181c* as compared to the negative control scramble. We observed ∼30% (p<0.01, t-test) decrease in viable cells in the *miR-181c* treated sample, relative to the negative control (Figure7A). This decrease could occur due to increased apoptosis, therefore we checked the activity of Caspase-3/7 in cells that were treated under the same conditions. There was ∼12% (p<0.05, t-test) increase in Caspase-3/7 activity in response to *miR-181c* upregulation relative to the negative control (Figure7B). These results suggest that the decrease of cell numbers with *miR-181c* addition could be partially explained by increased apoptosis. However, the level of apoptosis that we observed does not fully account for the much higher difference in the viable cell numbers, suggesting that *miR-181c* is also having an anti-proliferative effect which could be mediated through *p53* and *PCAF* (Figure8).

## Discussion

RNA from 12 brain tumor samples with some variation in their histological diagnoses was sufficient to detect a set of miRNA-mRNA pairs whose fluctuations are highly correlated. The merit of this statistical detection method is that it avoids non-physiological conditions required to alter gene expression. The number of such high correlation pairs was significantly greater than expected in the random case (Figure1). A weak positive correlative effect due to distance between the pairs could be detected (Figure4) as well as a weak negative correlative effect due to direct miRNA targeting of mRNAs (Figure2,3). However, among the very high correlation pairs only a small number bore a predicted target relationship (11 of 72, Table1) or were located on the same chromosome (7 of 72). For the remaining high correlation pairs the data does not imply any direction of casualty—is the correlation due to an effect of the miRNA on the mRNA or vice versa? Key to this approach was the endogeneous variations among the samples. In other words, the correlations might stem from individual differences and are not tumor specific. By restricting the samples to gliomas rather than including healthy controls, these data in general cannot directly address carcinogenesis. The interpretation of the correlations is limited to miRNA/mRNA relationships solely within our sample set.

Validation of the correlations was obtained by over-expressing or suppressing the implicated miRNA and measuring the mRNAs predicted to correlate. These experiments frequently matched the prediction and suggested that in these cases, the correlation was due to an effect of the miRNA on the mRNA (Table3). As expected, some pairs were not validated. Among the reasons for failure to validate are: (a) some of the high correlation pairs are false positives; (b) the U251 cells do not exactly replicate the tumors; (c) in some cases more than one miRNA contributes to the effect; (d) the direction of causality may pass from mRNA to miRNA; (e) direct causality does not underlie the relationship, rather the high correlation is the consequence that both the miRNA and the mRNA have a true biological relationship with a third element. Nevertheless, the statistical approach to endogenous fluctuations can reveal *in vivo* effects of miRNAs on the transcriptional profile.

**Figure 9:**
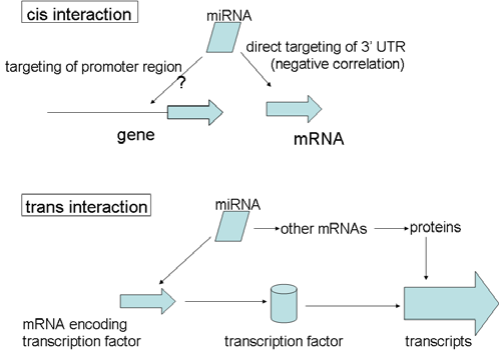
Mechanisms through which high miRNA-mRNA correlation pairs could operate.

Global effects of miRNAs upon the transcriptional profile and tissue specificity of mRNA expression have been reported [14], [15]. However, these studies have generally focused on miRNA targets which show more modest effects compared to the non-target effects described here. Furthermore, in one of the cited studies [15] the over-expression of a miRNA by transfection can create non-physiological changes upon the mRNA target field. Because direct miRNA targeting is unlikely in the absence of a seed region, as observed in most of the high correlation pairs, other explanations for how the high correlation pairs might arise were schematized (Figure9). Among the possible explanations for high correlation pairing is the binding of the miRNA to the promoter region of the paired gene with the result that transcription increases in those sets with positive correlations. A non-coding RNA binding to a promoter has been described [16], albeit in this case suppression occurred. Also possible is that the mRNA drives the effect on the miRNA.

Validation experiments allowed us to infer a tumor suppression pathway involving *miR-181c* (Figure8). *MiR-181c* and *PCAF* were among the endogenously highly correlated transcripts. We determined that *miR-181c* leads to an increase in *PCAF* and *p53* protein levels consistent with the known positive interactions between *PCAF* and *p53* (Figure6). At least two possible mechanisms could explain this result. The increase in *p53* protein levels could drive the increase in *PCAF* [12], [13]. Alternatively, the increase in *p53* could occur through *PCAF*, which was recently shown to stabilize *p53* by inactivating *MDM2*, an E3 ubiquitin ligase that negatively controls *p53* stability [17]. Concluding, the positive correlation of *miR-181c* with these two tumor suppressor genes can potentially explain the inhibitory effect of *miR-181c* on cell growth and the more subtle increase in apoptosis observed in the glioma cells (Figure8). Interestingly, *miR-181c* has been reported to be low in a panel of high-grade glioblastomas tumors and glioblastomas cell lines [18] and inhibition of another member of the *miR-181* family, *miR-181a*, in A549 lung carcinoma cells induced cell growth [19]. Lower levels of *miR-181c* in glioblastomas could indirectly decrease the transcript levels of the positively correlated *PCAF*, resulting in decreased activation of major tumor suppressive pathways in glioblastoma cells.

## Methods

### Sample preparation

Tissue from glioma resections were obtained and collected at UCLA under approval from the Institutional Review Board. Categorization into high or low grade was based on the formal pathology report, with WHO grades III and IV being termed “high grade” and grades I and II termed “low grade”. Histological diagnoses fell into the following categories: two low grade anaplastic mixed gliomas, one low grade oligodendroglioma, six high grade glioblastomas, two high grade gliosarcomas, and one high grade glioblastoma/gliosarcoma. From each the 12 tumor samples mRNAs and miRNAs were extracted.

### mRNA Microarrays

Microarray data were obtained from the 12 tumors using Affymetrics HG-U133A array platforms as described previously [20]. Briefly, total RNA was isolated from tumor samples using a TRIzol reagent (Invitrogen Life Technologies, Carlsbad, CA) and was followed by a cleanup on a RNeasy column (Qiagen, Hilden, Germany). cDNA was generated and cRNA probes were generated using standard protocols. Aliquots of each sample were hybridized to U133A oligonucleotide microarray (GeneChip Human Genome U133A, Affymetrix, Santa Clara, CA), which represents ∼14,500 human transcripts. The chips were scanned using the GeneArray scanner (Affymetrix). The CEL files generated by the Affymetrix Microarray Suite (MAS 5.0) were converted into DCP files using the DNA-Chip Analyzer (dChip 1.3, http://biosun1.harvard.edu/complab/dchip/). The processed data and the raw CEL files had been deposited in NCBI Gene Expression Omnibus database (series record GSE8692).

All arrays were performed using standard protocols recommended by the manufacturer within the UCLA DNA Microarray Facility. Briefly, all RNA samples had a 28S/18S ratio over 1.5 with no evidence of degradation. All scanned images were visually inspected for surface defects. Quality Control assessment of array data was performed using GCOS v1.4 (Affymetrix, Santa Clara, Ca) and Array Assist v5.0 build 30237 (Stratagene, La Jolla, Ca). Hybridization and Poly-A controls were within expected parameters, and 3′/5′ ratios for actin and GAPDH were below 3 (mean Beta Actin 3′/5′ = 1.53 and mean GAPDH 3′/5′ = 1.02). GCOS v1.4 Expression Reports also revealed expected Call percentages (Present: 48% to 62%; Absent: 36% to 49%; Marginal: 1.5% to 1.9%). All arrays were within 1.5 fold of each other in overall intensity.

### miRNA expression profile

Using the same 12 tumor samples from which the microarray data was obtained, miRNA profiling was performed by real-time multiplex RT-PCR [5]. Briefly, multiplexed reactions were performed with 330 sets of reverse transcriptase and second strand synthesis primers followed by individual singleplex TaqMan reaction in four 96-well reaction plates to measure the abundance of each of the 330 miRNAs after the multiplexed RT-PCR. Each primer and probe contained zip-coded sequences specifically assigned to each miRNA to increase the specificity of each reaction so that even small sequence differences in miRNA were amplified and detected. This approach is reliable as miRNA measurements on RT-PCR and microarray are concordant [21].

### Cell Culture and Reagents

U251 and U87 glioblastoma cells were plated out in DMEM containing 10% DMEM, 100 units/ml penicillin and streptomycin. Cells were cultured in a humidified incubator with 5% CO_2_ at 37°C. Transfections were performed using siPORT NeoFX (Ambion, Austin, TX). Transfection complexes were prepared according to the manufacturer's instructions and 1×10^5^/ml cells were added directly to the transfection complexes on the plate to a final pre-mir concentration of 50 nmol/L. The transfection medium was replaced 8 hours post-transfection. Three independent transfections were performed for each pre-mir and 2′*-O-*Me oligoncleotide of *miR-181c, miR-182, miR-19a* and a scrambled pre-miR or 2′*-O-*Me as a negative control (Ambion, TX).

### Real-Time PCR analyses of mRNAs

Total RNA was harvested 24 h after the transfection of U251 cells with the pre-mir by using the mirVana kit (Ambion) and 1.2 µg of total RNA was converted to cDNA by Superscript II first strand synthesis kit (Invitrogen, Carlsbad, CA), both according to manufacturers protocol. SYBR green real time PCR was performed by using the 7500 real-time PCR system (Applied Biosystems, Foster City, CA), POWER SYBR green master mix (Applied Biosystems) and gene specific primers for *PCAF, ASCC1, FADS2, HPS1, ANXA4, GSTO1, IFITM1, DDX11, TTC3, PRR13, ENTPD1, NCF2* and *GAPDH* (Applied Biosystems, http://www.allgenes.com). All the real-time PCR data was normalized to *GAPDH*. Melting temperatures for individual amplicons were determined, and proper PCR product sizes were confirmed using agarose gel electrophoresis. PCR assays were run in triplicate, and expression data were averaged. To calculate the relative expression for each gene, the 2^−ΔΔCt^ method was used.

### Western Blot

Total protein was isolated from U251 and U87 transfected cells in mRIPA cell lysis buffer. Protein concentration was measured using the BCA assay kit (Pierce, Rockford, IL). The membrane was first probed with antibodies against *PCAF* (H-369) (Santa Cruz Biotechnology, Santa Cruz, CA), anti-*p53* (BD Biosciences, San Jose, CA) and then with anti-beta-actin antibody (Sigma-Aldrich St. Louis, MO). Secondary antibodies were labeled with either Alexa Fluor 680 (Invitrogen) or IRDye800 (Li-Cor Biosciences, Lincoln, NE). Signals were visualized using the Odyssey infrared imaging system (Li-Cor Biosciences).

### Cell Growth and Apoptosis Assay

U251 cells were transfected with 50 nM of pre-*miR-181c* (Ambion) and 50 nM of negative control scramble (Ambion) and seeded in 96-well plates at 10,000 cells/well. Caspase-3/7 Glo (Promega, Madison, WI) reagent was used to determine the Relative Apoptosis 24 hours post-transfection. Cell Titer Glo (Promega) reagent was used to determine relative cell growth 24 hours post-transfection. Relative Apoptosis/Cell growth was determined by comparing *miR-181c* with scramble treated cells. Results represent the means of three separate experiments.

### Data processing

#### mRNA data

We computed the perfect match minus mismatch model-based expression values and performed quantile normalization implemented in dChip [22]. During normalization, we checked the single, array, and probe outliers such that these less reliable data were excluded. After normalization, we were able to compare the expression levels of all the genes in 12 samples. The presence-call was also determined for each probe set. This call indicates whether an expression level is reliable by labeling it as Present/Marginal/Absent through comparing the perfect match with the mis-match values for each probe set. Probe sets were linked to gene symbols using two annotation files, “HG_U133A_annot.csv” obtained from Affymetrix website and Ensembl (Release 43 February 2007) [23] through BioMart [24]. Due to the overlap between transcripts, a single probe set might detect more than one gene. Alternatively, different probe sets might be mapped into the same gene because many of them were designed to detect different splice variants of the same gene. We found that probes for the same gene usually showed very high correlation among themselves. For example, in the filtered data mentioned below, there were 2,563 sets of probes that detect the splice variants of the same genes. Probe sets that detected potentially alternatively splice variants were highly correlated with over 80% with correlation coefficients over 0.5.

The HG-U133A microarray chip contains 22,283 probe sets, from which a subset was selected for correlation analysis. We set up three criteria in selecting the subset: 1) to exclude less reliable data, the presence-call of a probe set through out 12 tumor samples had to be all Present. 2) to ensure high variability that permits the investigation of correlation, we selected genes which the maximum intensity among 12 samples was at least two fold higher than the minimum one. 3) a probe set had to have a gene symbol such that the biological relevance can be checked further. The rationale behind these criteria were: 1) Because correlation was critical to the analysis, we sought to reduce the uncertainty in the data as much as possible. In fact, when the level in one or more samples was labeled “Absent”, the overall levels in all samples were usually relatively low. Because low levels were usually accompanied with relatively high fluctuations, we tended to filter such cases. 2) The absence of high variability for a miRNA across samples implied that the observed variation was more likelyto be noise, and the resulting correlation spurious. 3) a gene symbol was needed for the target analysis and the output of highly correlated miRNA-mRNA pairs. 12542, 2547, and 105 probe sets were filtered away by criteria 1,2, and 3, respectively, and we were left with 7,089 probe sets for correlation analysis, which correspond to 5,822 distinct genes.

#### miRNA data

Multiplex RT-PCR data for miRNAs were normalized and reliability determined. The accuracy of measurement determinations with different amounts of miRNA samples demonstrated that a cut-off of 32 for the 30 ng sample was reliable [5]. Bias was clearly detected when comparing the levels of each miRNA from two samples before normalization (Figure S3). Without normalization, the fluctuation in overall concentration of miRNAs resulted in a strong artifactual auto-correlation among miRNAs themselves (Figure S4). Therefore, similar to the normalization of microarray data, quantile normalization on the multiplex RT-PCR results was performed. In brief, with N ( = 12 here) samples, each contains Ct values of m microRNAs, quantile normalization projects the N k^th^-tile's into the same value which is the average of the N k^th^-tile's (k = 1∼m). After normalization, the Ct distributions of the N samples overlap exactly by definition.

In order to increase the confidence, we selected a subset of miRNAs in which all the Ct's in 12 samples were reliable, i.e., less than the cutoff 32, and obtained Ct's for 94 miRNAs in each sample. This subset was then used for quantile normalization. After normalization, we further excluded one miRNA such that the remaining miRNAs all had strong signals, i.e., the maximum Ct among 12 samples was greater than the minimum by at least 1, a definition similar to the criteria used for mRNA data processing. The Ct's of these 93 miRNAs were then used for correlation analysis.

### Correlation coefficient and the random case

Before correlation analysis, we transformed the microarray intensity data into log scale of base 2 such that they were comparable with the Ct data of miRNAs. In addition, we converted a Ct value into 32-Ct such that the latter value was positively proportional to the log of copy number.

The distribution of correlation coefficients of two normally distributed random variables was utilized [25]. The derivation was recapped as follows: Given two profiles x_i_ and y_i_ with i = 1∼N, where N is the number of data points (N = 12 in our case), the Pearson's correlation coefficient r is defined as
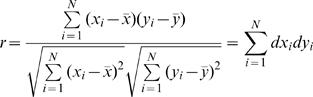
where dx_i_ and dy_i_ are the normalized profiles with mean equals zero and norm equals one (Σdx_i_ = 0 and Σdx_i_^2^ = 1). We can view dx_i_ and dy_i_ as two vectors on a uni-sphere in high dimensional space, then r is simply the inner product of these two vectors. For example, in three dimensions, without loss of generality we set z-axis as the direction of one vector, the inner product is simply the angle between the two vectors, i.e., r = cosθ. The probability density of r can be derived through 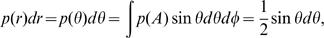
where A is the surface area in three dimensional space. For a random vector, the distribution on the surface should be uniform such that p(A) = 1/A = 1/4π, a constant. In n-dimensions, the probability density of θ is known as p(θ)∝sinθ^(n−2)^. Thus,
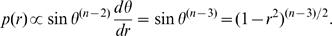
Since the vector is constrained to have its mean as zero, given N data points, the vectors stay in N-1 dimensions. Thus, n equals N-1 in the last equation; after normalization such that the probability adds up to one, we obtained the probability distribution as
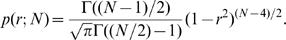



To confirm the probability distribution, we ran the following simulations. We used our miRNA/mRNA data and randomly generated a set of mRNA/miRNA data of the same size and compared the resulting distribution of correlations with the formula. All the 100 trials resulted in a distribution very close to the random case. In addition, we randomly permuted the sample order in the miRNA (or mRNA) profiles and repeated the analysis. The clear signals shown in figures of this work disappeared in all 100 such trials (data not shown).

### TargetScan prediction

Bulk data of the predicted mRNA targets of miRNAs in TargetScan (Release 3.1 November 2006) was downloaded from the website. We considered miRNAs in both conserved and non-conserved families (conservation across human, mouse, rat, dog, and sometimes chicken). The predicted targets only in human were extracted. Two binding sites were considered distinct if their seed region loci in the multiply aligned 3′UTR sequences were different. The 3′UTR length for each gene was determined from the 3′UTR sequence from TargetScan. We removed the masked bases and counted the remaining bases as the 3′UTR length.

### Estimation of slope in target percentage

We selected the correlation between −0.8 and 0.8 for slope estimation to avoid the large fluctuations due to small amount of miRNA-mRNA pairs outside the range. In Figure2, the slope of points inside this range was obtained by fitting the curve to a straight line.

### miRNA host genes and their loci

Host gene information was obtained from miRBase (Release 9.1 February 2007) [26]. Gene loci were obtained from Ensembl (Release 43 February 2007) [23] through BioMart [24] using dataset *Homo sapiens* NCBI36. Each gene loci is a range of base pairs and the distance between two genes was defined as the head to tail distance except when a miRNA is in the intron of an mRNA.

## Supporting Information

Figure S1 Relationship between the correlation of two miRNAs on the same chromosome strand and the distance separating the two miRNA loci (small points). The distances for all pairs of miRNAs were ranked, and the distance rank is used as the x-axis. For each distance rank, the physical distance on the chromosome strand in log-scale is shown (circles). To view the coarse-grained relationship, five correlations for each distance rank are averaged. For example, for distance rank 10, we averaged the five correlations of distance ranks 8 to 12.
(1.45 MB TIF)

Figure S2 Ratio of number of correlation coefficients between our experiments and the random case with 12, 11, 10, 9, 8, and 7 samples.
(1.45 MB TIF)

Figure S3 Scatter plots of the Ct values of two samples for all miRNAs. Two pairs of two samples were shown as an illustration.
(1.45 MB TIF)

Figure S4 Distribution of correlation coefficients among miRNAs before and after normalization.
(1.45 MB TIF)
