# Supplementary figures and images for: Detection of a MicroRNA Signal in an In Vivo Expression Set of mRNAs
Source: PLoS One. 2007 Aug 29;2(8):e804. doi: 10.1371/journal.pone.0000804 (PMC1950084; doi:10.1371/journal.pone.0000804)

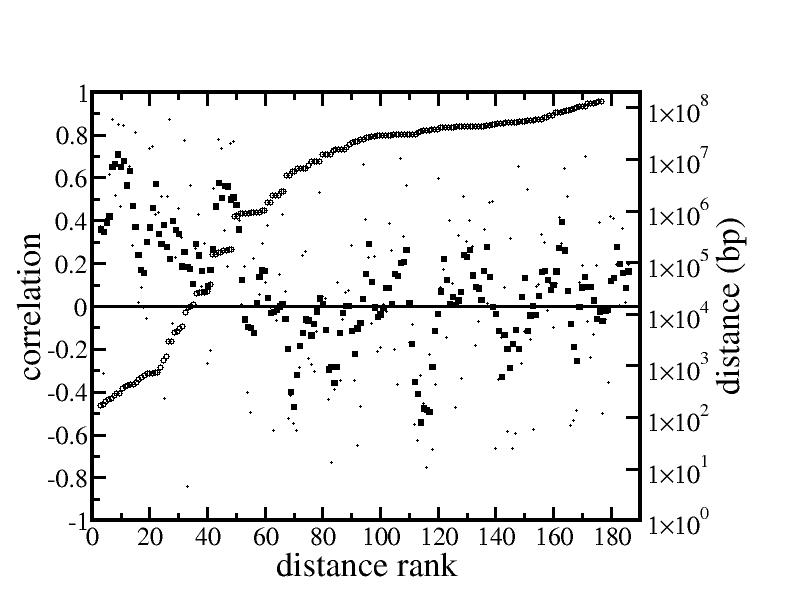

Supplement: Figure S1 — Relationship between the correlation of two miRNAs on the same chromosome strand and the distance separating the two miRNA loci (small points). The distances for all pairs of miRNAs were ranked, and the distance rank is used as the x-axis. For each distance rank, the physical distance on the chromosome strand in log-scale is shown (circles). To view the coarse-grained relationship, five correlations for each distance rank are averaged. For example, for distance rank 10, we averaged the five correlations of distance ranks 8 to 12. (1.45 MB TIF) [file pone.0000804.s001.tif]

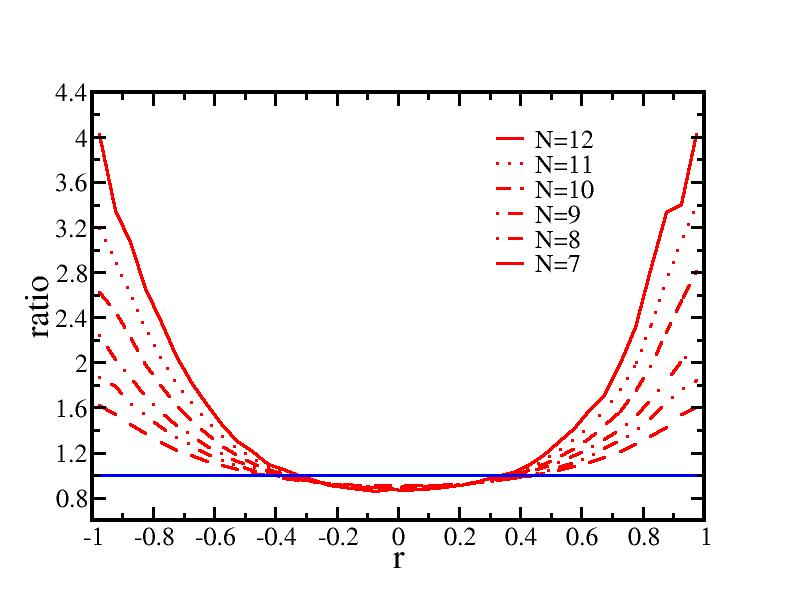

Supplement: Figure S2 — Ratio of number of correlation coefficients between our experiments and the random case with 12, 11, 10, 9, 8, and 7 samples. (1.45 MB TIF) [file pone.0000804.s002.tif]

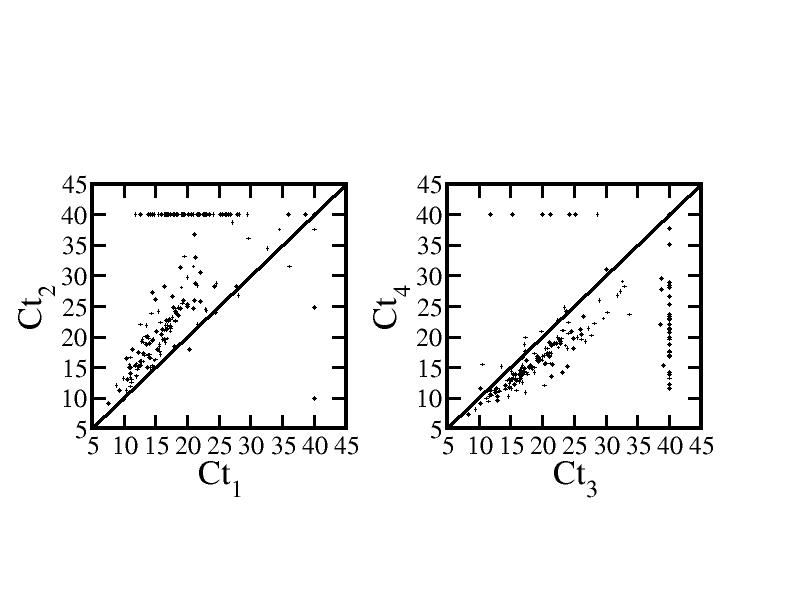

Supplement: Figure S3 — Scatter plots of the Ct values of two samples for all miRNAs. Two pairs of two samples were shown as an illustration. (1.45 MB TIF) [file pone.0000804.s003.tif]

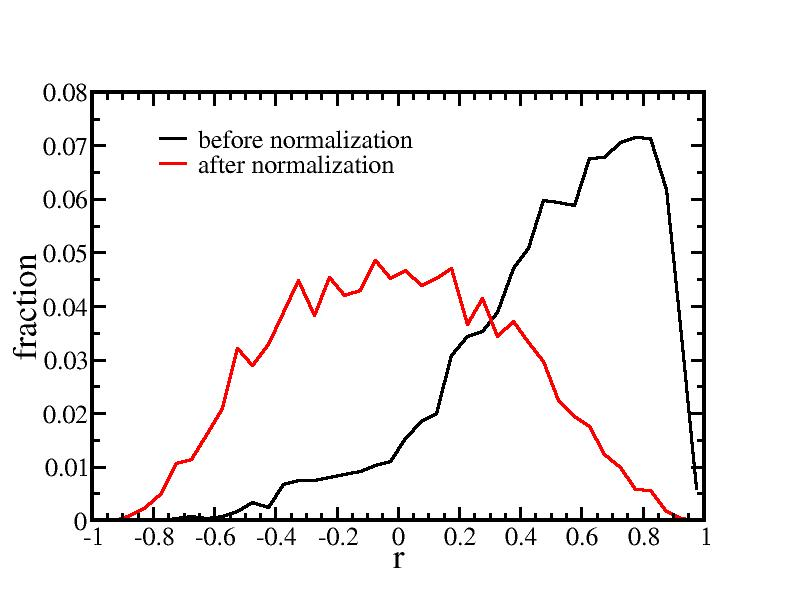

Supplement: Figure S4 — Distribution of correlation coefficients among miRNAs before and after normalization. (1.45 MB TIF) [file pone.0000804.s004.tif]
